# Feasibility of Producing Electricity, Hydrogen, and Chlorine via Reverse Electrodialysis

**DOI:** 10.1021/acs.est.2c03407

**Published:** 2022-10-18

**Authors:** Ameya Ranade, Kaustub Singh, Alessandro Tamburini, Giorgio Micale, David A. Vermaas

**Affiliations:** † Department of Chemical Engineering, Delft University of Technology, Van der Maasweg 9, 2629 HZDelft, Netherlands; ‡ Dipartimento di Ingegneria, Università degli Studi di Palermo, viale delle Scienze Ed. 6, 90128Palermo, Italy

**Keywords:** techno-economic assessment, levelized costs, upscale potential, salinity gradient energy, brine

## Abstract

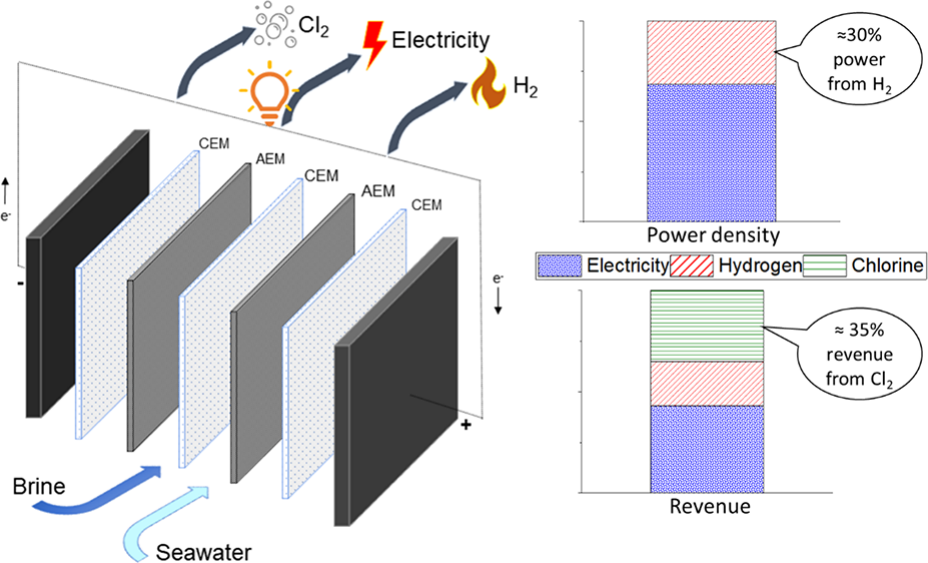

Reverse electrodialysis (RED) is a technology to generate electricity from two streams with different salinities. While RED systems have been conventionally used for electricity generation, recent works explored combining RED for production of valuable gases. This work investigates the feasibility of producing hydrogen and chlorine in addition to electricity in an RED stack and identifies potential levers for improvement. A simplified one-dimensional model is adopted to assess the technical and economic feasibility of the process. We notice a strong disparity in typical current densities of RED fed with seawater and river water and that in typical water (or chlor-alkali) electrolysis. This can be partly mitigated by using brine and seawater as RED feeds. Considering such an RED system, we estimate a hydrogen production of 1.37 mol/(m^2^ h) and an electrical power density of 1.19 W/m^2^. Although this exceeds previously reported hydrogen production rates in combination with RED, the levelized costs of products are 1–2 orders of magnitude higher than the current market prices at the current state. The levelized costs of products are very sensitive to the membrane price and performance. Hence, going forward, manufacturing thinner and highly selective membranes is required to make the system competitive against the consolidated technologies.

## Abstract 2

Mixing water streams with distinct salinity has the potential to produce renewable energy at scales relevant to the global energy consumption. We assess the techno-economic feasibility of combining electricity and hydrogen production via reverse electrodialysis.

## Introduction

The development of sustainable energy sources is crucial as the effects of greenhouse gases become evident. Although solar, wind, geothermal, hydro, and biomass are some of the proposed alternatives, each technology has certain bottlenecks and challenges. Salinity gradient power (SGP) is a technology that utilizes the salt concentration differences between two water streams to generate energy. This can be applied throughout the world wherever two streams with different salinities are available in the same location (e.g., freshwater streams flowing into the sea or brine streams close to low salinity wastewaters). The global potential of SGP using natural feed waters is over 2 TW, which represents approximately 20% of the worldwide energy demand.^1^ Reverse electrodialysis (RED) is a technique able to convert SGP directly into electricity and has received growing attention in recent years. An RED stack typically contains alternating anion exchange membranes (AEMs) and cation exchange membranes (CEMs), which are the core of the technology. The membranes are separated by spacers that form the channels where the two streams are forced to flow. The cell pair composed of an AEM, a CEM, and two spacers is the repeating unit of the RED stack. When a high salinity solution (HSS) and a low salinity solution (LSS) flow into these compartments, anions and cations in the HSS are transported to the adjacent LSS through the AEMs and CEMs, respectively. This movement of ions results in a separation of charge, thus generating an electric potential. These ionic fluxes are converted into electricity at the end compartments of the stack where the electrodes are present. Here, redox reactions occur via the redox-active electrode rinse solution (ERS), generating the electrical current that flows in the external circuit once an external load has been connected.

Recent developments in RED such as the use of profiled membranes^2^ and electrode segmentation^3^ have improved the overall performance of the system. The research focusing on the impact of RED feeds,^4^ membrane permselectivity,^5^ concentration of multivalent ions in RED feed solutions,^6^ use of capacitive electrodes,^7^ and co-ion and osmotic transport^8,9^ has provided a deeper understanding and facilitated the construction of prototypes and pilot plants from the lab scale.^10,11^

While RED has been conventionally used for electricity generation, a part of the obtained energy is utilized at the electrodes to perform the redox reactions. Hence, various alternatives for electrode rinse solutions in RED have been proposed.^12−14^ To minimize energy losses at the electrodes, reversible redox couples such as [Fe(CN)_6_]^3–^/[Fe(CN)_6_]^4–^ and Fe^2+^/Fe^3+^ have been used owing to their favorable characteristics such as good conductivity and low overpotentials. However, due to the imperfect nature of membranes, precipitation of salts in electrode compartments can take place by transport of co-ions from the feed compartments to the electrode compartments. In addition, a possibility of gas evolution exists while using these solutions.^15^ Hence, a suitable combination of RED feeds and ERS becomes essential.

An alternative of overcoming the ERS limitations is by adapting the system to deliberately produce valuable gases from the electrode reactions. Hydrogen’s high energy density, benign nature, and promising choice as a sustainable energy carrier^16^ have prompted research in combining RED and hydrogen production.^17−20^ Various aspects of the combined RED + H_2_ systems such as the use of microbial fuel cells,^21−24^ waste acid neutralization,^25^ and the use of acid and base as the electrode solutions^16^ have already been investigated. The practical potential of this combination using ammonium bicarbonate solutions as RED feed has been assessed as well.^26,27^Table 1 provides a comparison of hydrogen production rates, RED feeds, and ERS used for combined RED + H_2_ systems currently present in literature.

**Table 1:** Comparison of Various RED + H_2_ Systems Present in Literature

ref	cell pairs	H_2_ production (mol/(m^2^ /h)	RED feeds		ERS	
			HSS	LSS	catholyte	anolyte
(21)	5	0.13	1.4 M NH_4_HCO_3_	H_2_O	1 M NaHCO_3_	1 g/L NaOAc Buffer
(22)	7	0.11	1.7 M NH_4_HCO_3_	22 mM NH_4_HCO_3_	1 M NaHCO_3_	1 g/L NaOAc Buffer
(25)	20	0.06	1.4 M NH_4_HCO_3_	5 mM NH_4_HCO_3_	0.01 M HCl	1.4 M AmB
(16)	20	0.24	4 M NaCl	17 mM NaCl	0.5 M HCl	0.5 M NaOH
(28)	100	1.1	0.5 M NaCl	17 mM NaCl	H_2_O (pH ≈ 6)	
(26)	10	0.72	2 M NH_4_HCO_3_	0.06 M NH_4_HCO_3_	1 M KOH	

When considering integrated RED + H_2_ systems for large-scale applications, two practical challenges appear: (i) the typical current density of RED is 1–2 orders of magnitude lower than that of water electrolysis that results in a lower hydrogen production compared to a conventional water electrolyzer, thus decreasing the output from the system, and (ii) chlorine evolution competes with oxygen evolution at the anode. The latter may be suppressed by using Na_2_SO_4_ solution as the ERS, but that will not be sustained when using NaCl-rich feed (such as brine or seawater) and when using membranes that are never perfectly selective.^28^ Hence, frequent replacement or refining of the ERS will be necessary.

The disparate current density of RED and water electrolysis poses a serious engineering challenge. Although seawater and river water are the most common feeds for RED, the 1–2 orders of magnitude disparity in current densities cannot be bridged by minimizing the intermembrane distance^29^ and pre-blending the river water.^30^ A rough scan of the maximum obtainable current density reveals that the current density in the case of RED fed with brine and seawater is about 5 times higher than in RED fed with seawater and river water (Supporting Information, Figure S1). Even though the obtained power densities are greater in the case of feeding brine and brackish water^4,31^ and the electromotive force is higher while using seawater and river water,^32^ the higher conductivity and greater abundancy of brine and seawater (when comparing with brine and brackish water) outweigh these effects when considering an RED system combined with hydrogen production.

**Figure 1:**
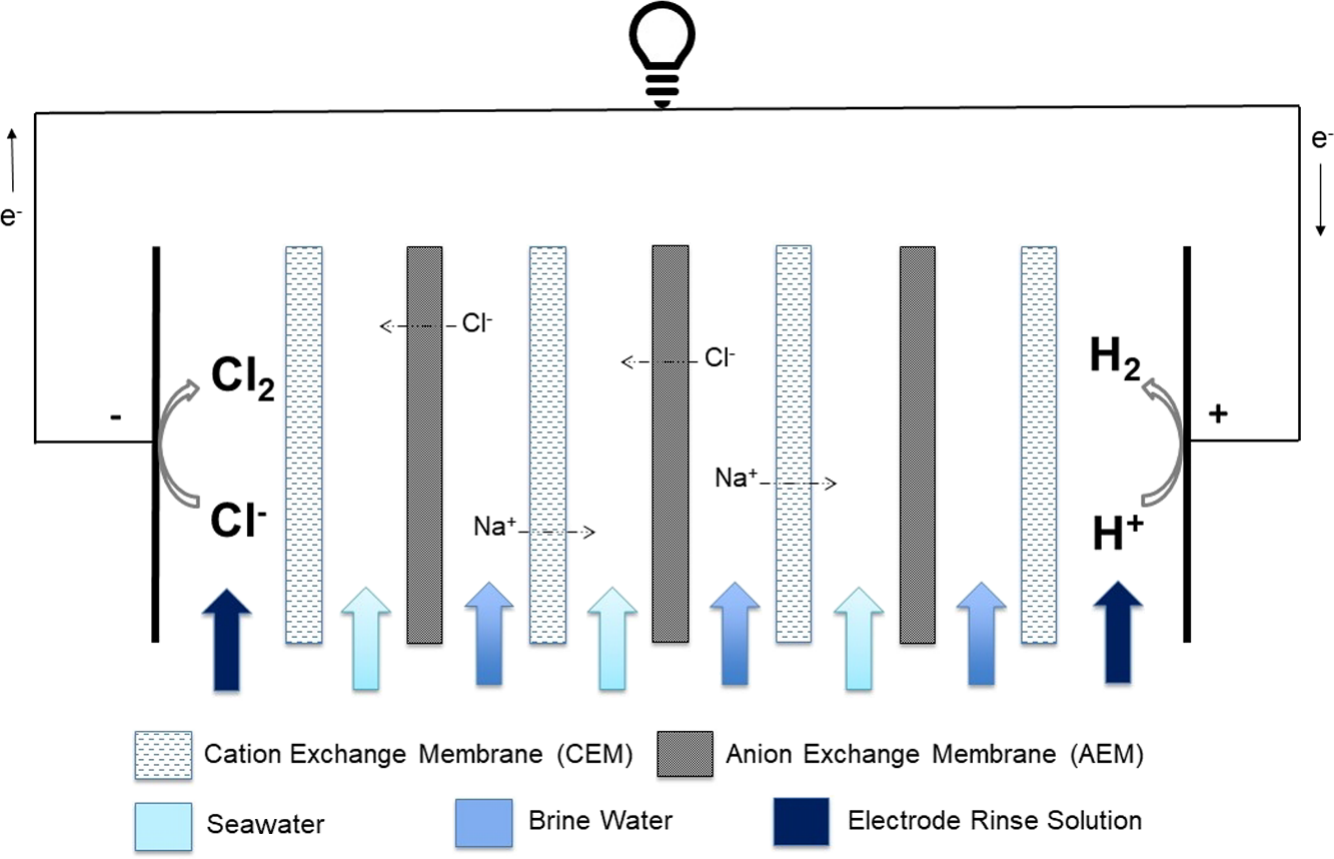
Schematic representation of the investigated reverse electrodialysis (RED) system combined with hydrogen and chlorine production. Brine water (BrW) and seawater (SW) are used as RED feeds, and acidified brine water is the electrode rinse solution (ERS).

Therefore, to apply combined RED + H_2_ systems at a large scale, we assess the following strategy: combining RED fed by brine and seawater, paired with hydrogen and chlorine production. The proposed system (schematically presented in Figure 1) possesses several advantages as compared to a conventional RED system fed by seawater and river water. (i) The use of brine and seawater drastically increases the conductivity of the combined system, requires lower purification costs, avoids competition with fresh water use, and is potentially less susceptible to fluctuations in concentrations.^31^ (ii) Accepting chlorine as a product while using chloride-rich streams such as brine eliminates the need for refining the ERS and provides additional economic potential. Using brine and seawater, and adopting chlorine as a product, mitigates the two bottlenecks in combined RED + H_2_ technology, namely, the relatively low current densities, and Cl^–^ containing natural feeds. Hence, we argue that the economic feasibility of a combined RED + H_2_ + Cl_2_ system is equal or better than RED + H_2_, and thus, it represents a useful case for improving the system’s economic assessment.

The present work aims to evaluate the practical potential of a combined RED + H_2_ + Cl_2_ system wherein the effect of producing chlorine in addition to electricity and hydrogen will be considered for the first time. To do so, a simple techno-economic model (see the next section) for the RED + H_2_ + Cl_2_ stack system is adopted and a number of simulations are presented. The relevant parameters are introduced first followed by the equations that describe the modeled system. The results consider the impact of varying numbers of cell pairs, membrane price, resistance, permselectivity, and discount rate. Their effects on the power density and the levelized cost of products are discussed in the technical analysis and the economic analysis, respectively. This is followed by a sensitivity analysis, which determines the influencing parameters in the proposed system. This work reveals the feasibility of combining RED with hydrogen and chlorine production and identifies potential levers to improve its economic feasibility.

## Techno-Economic Model

**Figure 2:**
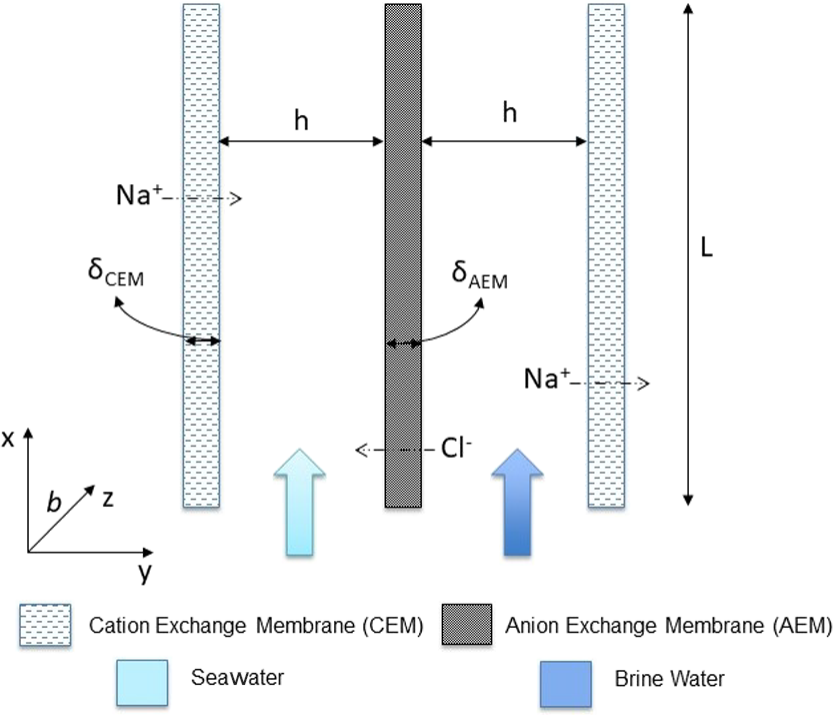
Schematic of an RED cell pair. The cell pair constitutes a cation exchange membrane (CEM) and an anion exchange membrane (AEM) along with the two spacers separating them. Brine and seawater flow in the concentrate and the diluate compartments.

The system modeled in this work consists of an RED stack composed of several cell pairs. The cell pair is the smallest repetitive unit of the stack and forms the basis upon which the model and the techno-economic analysis are constructed. A single cell pair (schematically represented in Figure 2) consists of an AEM and a CEM, along with two spacers between the membranes, which form the inlet compartments. For the present case, brine water (BrW) and seawater (SW) flow through alternate compartments. The RED feeds flow parallel to the membranes (along the *x* axis in Figure 2). Application of current results in the movement of ions from the concentrate to the adjacent diluate compartments in the direction perpendicular to the membranes. The hydrogen and chlorine gases are evolved from the electrode compartments. The pH of the ERS is assumed to be 2 to (a) reduce chlorate, hypochlorite, formation, etc.,^33^ and (b) to achieve acidic conditions, which have been known to favor hydrogen production.

The focus of the present work is providing a preliminary feasibility analysis of RED systems producing electricity, hydrogen, and chlorine. To achieve this, a simplified one-dimensional model (modeling along the main flow direction) has been developed in MATLAB that takes into account all the relevant phenomena such as ion fluxes, power obtained, gas produced, etc. These are presented in the following sections. All the parameters describing the system and adopted for the simulations are presented in Table 2. The model adopted is also based on the following simplifying assumptions:

- The feed streams are modeled as water-NaCl solutions (i.e., the presence and influence of multivalent ions are neglected).
- Contributions from the boundary layer phenomena (non-ohmic effects) are neglected, which is reasonable for high feed concentrations.^4^
- Uniform flow distribution is assumed across all compartments.
- Selectivity of chlorine at the electrode is 90%.^34^
- Membrane resistance is proportional to its thickness.
- Ionic short-circuit currents are ignored.
- Perfect gas–liquid separation is assumed.
- Gas evolution at the electrodes does not generate additional mass transport resistances.
- Variables are discretized along the main flow direction (*x* direction). The degree of discretization was left to its default value, usually set by the ode solver. This was found to be more than sufficient to avoid any residual numerical effects.
- Salt diffusive transport and water transport across IEMs (i.e., osmosis and electro-osmosis) are neglected.

**Table 2:** Technical and Economic Data Used as Model Input

parameter	symbol	default value	range	reference
Technical parameters				
length of the compartment (m)	*L*	1		(31)
width of the compartment (m)	*b*	1		(31)
intermembrane distance (μm)	*h*	300	100–500	
brine water residence time (s)	*t*_res,HSS_	80^b^		
seawater residence time (s)	*t*_res,LSS_	80^b^		
temperature (K)	*T*	298.13		
number of cell pairs	*N*	100	50–200	
permselectivity (for both the AEM and CEM)	α	70%	70%, 95%	
brine water concentration (M)	*c*_HSS_	5		
seawater concentration (M)	*c*_LSS_	0.5		
anion exchange membrane resistance (Ω cm^2^)	*R*_AEM_	1.4	0.7–2.8	(35)
cation exchange membrane resistance (Ω cm^2^)	*R*_CEM_	0.6	0.3–1.2	(35)
membrane thickness (wet) (μm)	δ_AEM/CEM_	80	40–160	
spacer porosity	ε	0.7		(36)
spacer shadow factor	β	0.5		(36)
Economic parameters				
RED casing (€/m^2^_electrode_)		2		(31)
electrodes (€/m^2^_electrode_)		500		(31)
spacers (€/m^2^_membrane_)		1		a
membranes (€/m^2^_membrane_)		50	5–100	a
membrane lifetime (years)		10		a
plant lifetime (years)		20		a
annual working hours		7200		a
discount rate	*r*	5%	1–6%	a
construction (€/m^2^)		1123		(37)
pumps and equipment (€/kW)		1600	800–2400	(37)
filtration (€/kW)		1850	925–2780	(37)
labor		20% of CAPEX	10–30%	(37)
OPEX (annual)		6% of CAPEX	4–8%	(37)

a Assumption.

b Velocity in the channels = 1.25 cm/s.

The RED feed streams of the proposed system have a high salt content, and thus, the physiochemical properties deviate from ideal conditions. The activity coefficients consider the deviations from ideal behavior, which become important in the case of high salt concentrations. These are estimated using Pitzer’s correlations.^38^ Other physical properties of solutions such as conductivity and viscosity are estimated from the correlations presented in ref (39).

In an RED stack, the primary mode for the generation of current and voltage is the transport of ions through the membranes. Mass transport through an IEM takes place from the concentrate to the diluate compartment. The molar flux (*J*(*x*)) is given by
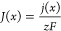
1where *j*(*x*) is the local current density (A/m^2^), *z* is the valence of ions (*z* = 1 for NaCl solutions), and *F* is Faraday’s constant (96,485 C/mol).

This transport of ions changes the concentrations in the compartment along the streamwise direction (*x*). These can be calculated via the following mass balances.^40^
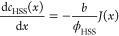
2
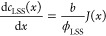
3where ϕ_HSS_ and ϕ_LSS_ are the volumetric flow rates (m^3^/s) of RED feeds and *b* is the compartment width.

A potential is developed across the membranes because of the differences in ionic concentrations in each compartment. In the absence of current, this potential is simply referred to as the open circuit voltage (OCV). When multiple membranes are stacked together, the voltage over these membrane pairs accumulates. This overall potential can be expressed in the form of the Nernst law by
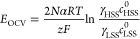
4where *E*_OCV_ is the OCV (V), *N* is the number of membrane cell pairs, α is the permselectivity of membranes, *R* is the universal gas constant (8.314 J/(mol K)), *T* is the temperature (K), γ is the activity coefficient, and *c*^0^ is the bulk inlet concentration on each side (mol/m^3^).

The local electromotive force that varies along the flow direction is given by
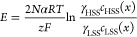
5When an RED stack is connected to an external load, the voltage experienced can be given by the difference in the total electromotive force and the voltage drop across the stack resistance.
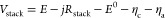
6where *V*_stack_ is the voltage over the stack, *j* is the current density (A/m^2^) (varied between the short-circuit conditions, representing maximum driving force to open circuit, which represents minimum driving force^3^), *R*_stack_ is the stack resistance (Ωm^2^) (see eq 7), *E*^0^ are the standard reaction potentials for hydrogen and chlorine evolution, and η_c_ and η_a_ are the overpotentials for hydrogen and chlorine evolution, obtained from refs (41) and (42), respectively.

In an RED unit, the resistances compose of the membranes, spacers, electrodes (other equipment such as wires, connections, etc.), and HSS/LSS compartments. Their contributions can be estimated by the following equation:^36^
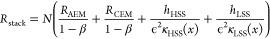
7where *R*_AEM_ and *R*_CEM_ are the area resistances of AEMs and CEMs (Ωm^2^), respectively, β is the spacer shadow factor, *h* is the intermembrane distance (m), ϵ is the spacer porosity, and κ(*x*) is the local electrical conductivity (S/m).

Energy is required to pump the solutions through an RED cell. The amount of energy required can be calculated from the pressure drop across the compartments and the flow rate of the feed solutions. Assuming equally thick compartments, the pumping power required for one cell pair can be calculated by^36^
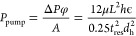
8where Δ*P* is the pressure drop (Pa), *A* is the total membrane area, φ is the flow rate (m^3^/s) in one compartment, μ is the viscosity of feeds (Pa·s), *L* is the compartment length (m), *h* is the intermembrane distance (m), *t*_res_ is the residence time (s), and *d*_h_ is the hydraulic diameter (m), calculated from the equations described in ref (36) where the spacer filaments are taken into account during calculations.

In the present case, the gross power obtained from the system comes directly from the generation of electricity and indirectly from the production of hydrogen, when the latter is considered as an energy source.^43^ The contribution of electrical power density can be calculated by multiplying the stack voltage by the current density.
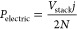
9

The hydrogen produced is calculated by Faraday’s law:
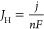
10where *J*_H_ is the flux of hydrogen produced (mol/m^2^/s). The hydrogen equivalent power density (per m^2^ of membrane) is calculated by multiplying the hydrogen produced by its higher heating value (HHV) (285.8 kJ/mol).
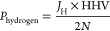
11

The gross power density is obtained by adding the contributions of electrical and hydrogen power.
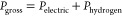
12

The net power density is then calculated by subtracting the pumping power from the obtained gross power
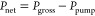
13

The levelized cost of energy is a useful economic indicator to compare the prices of products from different sources. The expression to calculate the levelized costs is shown below
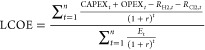
14
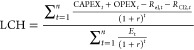
15
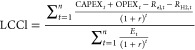
16

Here, capital expenditure (CAPEX_*t*_) and operating expenditure (OPEX_*t*_) are the expected expenditures in year *t*, *E*_t_ is the estimated production of the respective products, *n* is the expected lifetime of the plant, and *r* is the discount rate. While calculating the levelized costs of each product, the revenue generated from the other products is subtracted from the incurred costs so that double counting is avoided. For example, while calculating levelized costs of electricity (LCOE), the revenue obtained from hydrogen (*R*_H2_) and from chlorine (*R*_Cl2_) is subtracted from the incurred costs. The default prices of each product are considered as electricity: 0.1 €/kWh,^44^ hydrogen: 4 €/kg,^45^ and chlorine: 0.2 €/kg.^46^ The size of the RED system modeled here was constrained by the total amount of brine that could be used for electricity and hydrogen/chlorine production. This was done to keep the operation realistic. The global production of brine (obtained from rock salt mining, desalination streams of RO plants, and salt factories) is roughly 280 million tons per year,^47,48^ with the chlor-alkali process having a major share (≈39%).^49^ As a consequence, the annual brine consumption of a chlor-alkali plant in Europe (approximately 200 kton/year^50^) was used as a benchmarking tool. Hence, we assume that the modeled RED system consumes ≈1% of the brine currently used in Europe’s chlor-alkali industry (i.e., 200 kton salt per year), which completes the boundary conditions for the simulations.

## Results and Discussion

The following section discusses the influence of various technical and economic parameters on the performance of the process. Particular attention is given to the power density in the case of technical analysis and the levelized costs of products in the case of economic analysis as they are simple metrics for comparison. All results considered in this section are calculated for default values given in Table 2, unless otherwise stated.

### Technical Analysis

**Figure 3:**
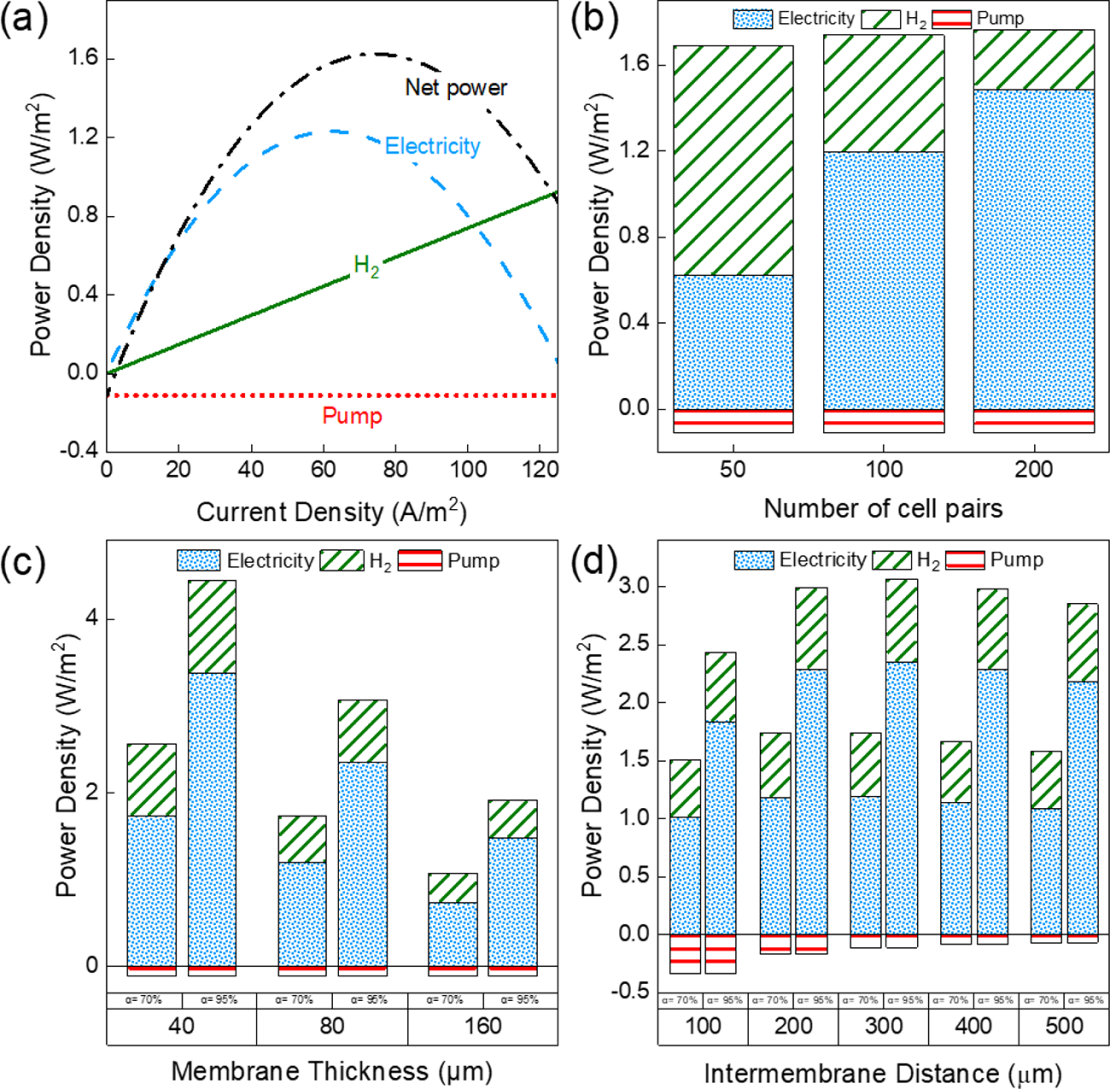
Power density of a combined RED + H_2_ system plotted as a function of (a) current density (for 100 cell pairs), (b) number of cell pairs, (c) membrane thickness, and (d) intermembrane distance, calculated under conditions where the produced net power density is maximum.

Figure 3a shows a plot of the power densities against current density. It must be noted that the pumping power density is independent of the current density (eq 8). The contribution of chlorine is not considered here as it is not considered as an energy carrier. Rather, its production is used as a revenue in the economic analysis. It is evident from Figure 3a that when power obtained from hydrogen is included in the net power calculation, the maximum point shifts right from the maximum of the net power density when only electrical power density is considered. This shifted point represents the maximum output between the amount of hydrogen and electricity produced. Using the default values from Table 2 and Figure 3a, a hydrogen production of 1.37 mol/(m^2^ h) (0.54 W/m^2^ membrane) and electrical power density of 1.19 W/m^2^ are obtained where the net power density exhibits a maximum. The total gross power of the combined RED + H_2_ system is 45% larger than the (gross) power in the form of electricity only, and the net total power (including pumping power) is 50% larger than the net electrical power only. Hence, the chemical energy of the produced hydrogen is significant, even when using 100 cell pairs.

By tuning the current density, the ratio between electricity and hydrogen can be varied in response to demand (e.g., during times of low electricity demand, the system can be set to store energy in the form of hydrogen production by increasing the current density and operate closer to short-circuit conditions). This flexibility in varying the ratio between electrical power and hydrogen production is only possible when a substantial voltage is generated by the RED cells. For example, the hydrogen and chlorine evolution reactions, including reaction overpotentials, would require an OCV of ≈1.7 V, corresponding to approximately 20 RED cell pairs using brine water and seawater as RED feeds. The use of a substantial number of RED cell pairs also makes the system more resilient for fluctuations in feed supply or feed water concentration; a low number of RED cells face the risk of not exceeding the threshold for hydrogen and chlorine evolution when feed conditions are sub-optimal. Hence, as opposed to the previous works reported in Table 1, we argue that using a substantial number of RED cell pairs (>20) yields more promising business cases.

The number of cell pairs in a stack also influences the contributions of hydrogen equivalent power density and electrical power density. Figure 3b shows the contributions of hydrogen, electrical, and pumping power densities for 50, 100, and 200 cell pairs, respectively. When considering 50 cell pairs, the contribution of hydrogen equivalent power density is high (1.06 W/m^2^) since the majority of the voltage generated over the stack is spent at the electrodes to perform the redox reactions. Consequently, the amount of energy that can be utilized as electricity is low (0.6 W/m^2^). As the cell pairs increase, the voltage generated across the stack increases. However, the hydrogen produced remains almost the same as it is dependent on the current flowing through the circuit. Hence, when normalizing over the surface area, as cell pairs increase, the contribution of electrical power density increases (1.19 W/m^2^ for 100 cell pairs, 1.48 W/m^2^ for 200 cell pairs) while the contribution of hydrogen equivalent power density decreases (0.54 W/m^2^ membrane for 100 cell pairs, 0.27 W/m^2^ for 200 cell pairs). The pumping power density remains the same (−0.11 W/m^2^ (denoted negatively as it is deducted while calculating the net power density)) since it is normalized by the membrane area and remains proportional to the number of cell pairs.

To improve the power density, the thickness of the ion exchange membranes is an important lever. This effect is shown in Figure 3c where the power densities are plotted against the membrane thickness for two different membrane permselectivities, i.e., 70 and 95%. Two main conclusions can be drawn from this plot. First, if the permselectivity is kept constant, then the net power density increases by approximately 50% when the membrane thickness is halved (e.g., 1.62 W/m^2^ for 80 μm vs 2.44 W/m^2^ for 40 μm, at 70% permselectivity). Second, the net power density of highly selective thick membranes is slightly increased compared to that of current thin membranes (e.g., 1.8 W/m^2^ at 95% permselectivity, 160 μm vs 1.62 W/m^2^ at 70% permselectivity, 80 μm). Increasing the membrane thickness will increase the permselectivity because water transport will be limited. These findings indicate that there are two primary ways to increase the power density from the system: first is the reduction in membrane thickness, which typically decreases the membrane resistance and thus increases the power output. Second is the increase in membrane thickness and permselectivity, which will also increase the power output.

The intermembrane distance can also affect the net power output of the cell. As opposed to cases using very dilute feed water,^29^ the generated power is not very sensitive to the intermembrane distance in our case due to the already highly conductive feed. Figure 3d shows the power densities for intermembrane distances from 100 to 500 μm at 70 and 95% permselectivities. The pumping power density and the compartment resistance are influenced by the intermembrane distance, which in turn impacts the net power output of the cell. Increasing the intermembrane distance leads to higher electrical resistance (lower gross power) and lower velocity (lower pumping power). These result into an increasing–decreasing behavior, exhibiting a maximum. As a consequence of the two, the net output from the stack reduces when deviating from the optimal spacer thickness, in this case, close to 300 μm. The exact optimum depends on the feed water and membrane properties; for example, a less conductive seawater/river water feed will lead to a lower optimal intermembrane distance. In our case, the obtained total power output is more sensitive to the membrane properties, such as membrane resistance (tuned by the thickness; Figure 3c) and apparent permselectivity, rather than the intermembrane distance (Figure 3d). Comparing the performance of highly selective membranes to current membranes while keeping other parameters constant, the stack voltage and the current density corresponding to the maximal point of hydrogen and electricity power increase by at least 30% when using highly selective membranes.

### Economic Analysis

**Figure 4:**
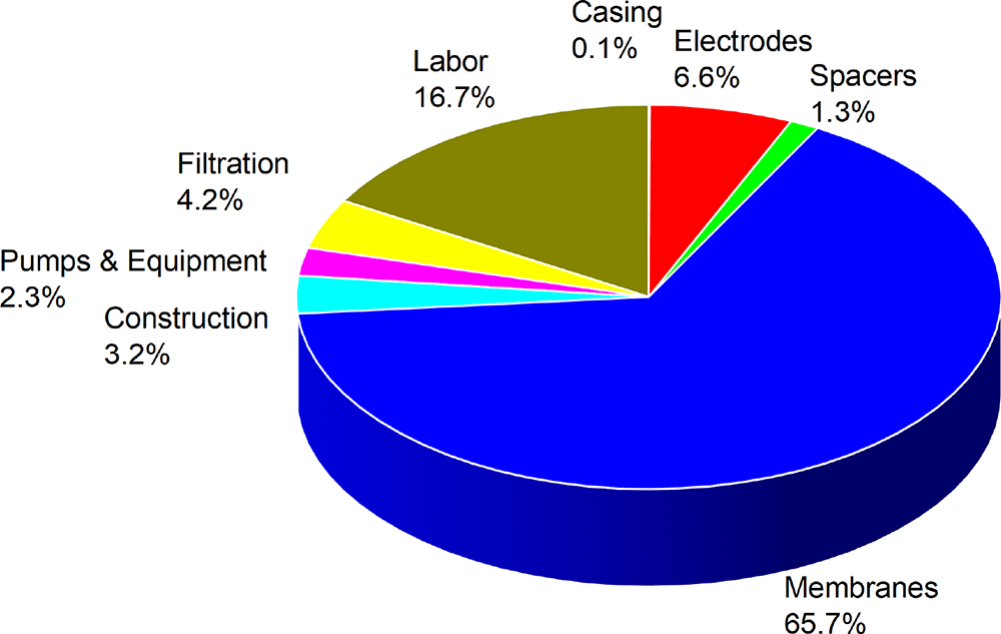
CAPEX contribution of the system using the default values from Table 2.

The feasibility of an RED + H_2_ + Cl_2_ system greatly depends on the marketability of the products. Here, the levelized costs of products can give an impression as to at what prices the products need to be sold to achieve the required rate of return. Using the default values in Table 2, the levelized costs of products are presented in Table 3 and the contribution of various parameters in the CAPEX is highlighted in Figure 4. We can clearly see that the levelized costs are 1–2 orders of magnitude higher than the current market prices, and hence, it is necessary to tune the parameters to achieve feasibility. This is discussed in the following section.

**Table 3:** Levelized Cost of Products Using Default Values from Table 2

product	levelized cost
electricity	1.5 €/kWh
hydrogen	117 €/kg
chlorine	3.7 €/kg

**Figure 5:**
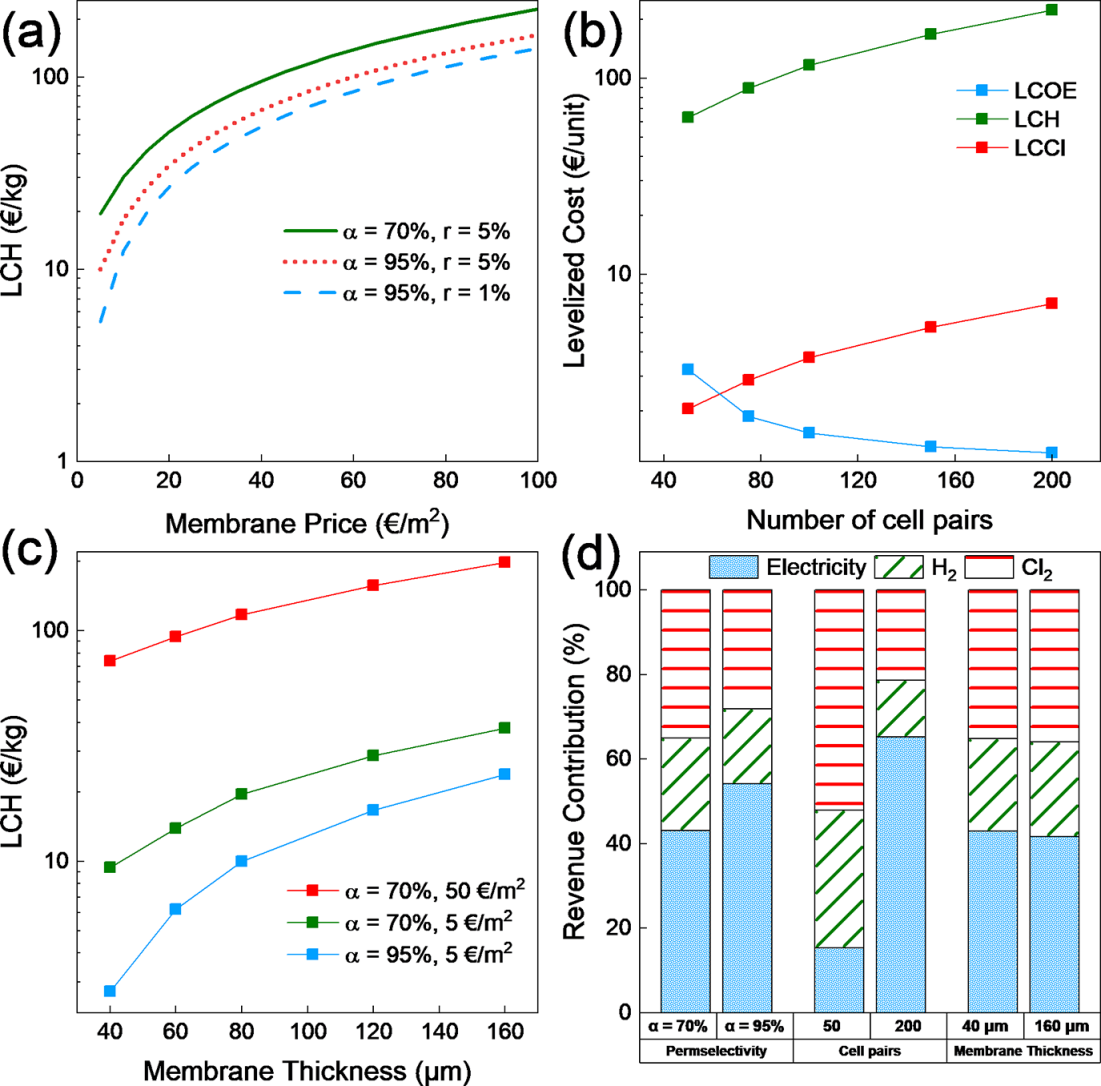
(a) Levelized cost of hydrogen (LCH) plotted against the membrane price, (b) levelized cost of products vs the number of cell pairs, (c) levelized cost of hydrogen against membrane thickness, and (d) revenue contribution (%) against various parameters.

The membrane price is possibly the most crucial factor when considering economic feasibility since it accounts for roughly 60% of the total costs. The impact of the membrane price on LCH is discussed in Figure 5a, and it is observed that the LCH increases with an increase in the membrane price under all scenarios. Assuming a current membrane price of 50 €/m^2^, the LCH comes out to be 117 €/kg, which is almost 30 times more than the current market price (4 €/kg). Even if the membrane prices are reduced to 5 €/m^2^, the LCH decreases to 19.5 €/kg and falls short of competing with the market prices. Consequently, the use of highly selective membranes (95% permselectivity) and reduction in discount rate is also considered to observe the improvement in LCH. Using highly selective membranes (95% permselectivity), the LCH reduces by approximately 50% at all membrane prices. However, even in this case, at a membrane price of 5 €/m^2^, the LCH comes out at 10 €/kg and remains above the market price. Going forward, the discount rate is also reduced to 1% in addition to high permselectivity. The discount rate refers to the rate of interest that is applied to the future cash flows of an investment to calculate its present value. A low discount rate indicates a low return on the process since future cash flows are discounted at a lower rate. In this case, the LCH (5 €/kg) is comparable with the current market prices when the membrane price is 5 €/m^2^. A favorable LCH at low discount rates also indicates that even if this technology is commercialized, the rate of return will be low. The levelized costs of electricity and chlorine as a function of the membrane price are given in Figure S2.

Alternatively, the number of cells pairs and membrane thickness can be adapted. Variation in the number of cell pairs not only changes the proportion between electricity and hydrogen production but also affects the levelized cost of products. This effect is shown in Figure 5b. The LCOE decreases as the number of cell pairs increases (3.2 €/kWh for 50 cell pairs to 1.2 €/kWh for 200 cell pairs). This is primarily because the stack voltage is proportional to the number of cell pairs. We note that this relation does not hold for a large number of cell pairs due to ionic short-circuit currents, which are ignored in the model presented here. A higher number of cell pairs generate a higher voltage, which makes the energy consumption for the redox reactions relatively small and leads to more electricity production and lower costs per kWh. The hydrogen or chlorine produced is dependent only on the current flowing through the circuit and does not change significantly when changing the cell pairs. This translates to a higher cost with an increase in cell pairs (63 €/kg for 50 cell pairs to 223 €/kg for 200 cell pairs, for hydrogen, keeping default values for the membrane price and performance). The variation in levelized costs changes by a factor of 3 and can affect the economic viability of the process. Hence, the number of cell pairs is an important parameter to be considered during detailed designing and deciding between pure RED vs RED + hydrogen production.

The membrane thickness also affects the levelized costs, as shown in Figure 5c. A thinner membrane leads to higher electricity/hydrogen production, which in turn reduces the levelized costs of products. The LCH decreases by approximately 50% when the membrane thickness is halved. However, even in this case, the membrane prices affect the LCH significantly. For a thin membrane (40 μm), the LCH reduces from 74 to 9 €/kg by reducing the membrane prices from 50 to 5 €/m^2^. Subsequently, if the effect of permselectivity is also considered, on top of using thinner membranes and reduced membrane prices, then the LCH (2.7 €/kg) can compete with the current market prices. We realize that it is difficult to quantitatively estimate the effect of thickness on the membrane price, and hence, we assume the membrane price to be independent of membrane thickness. The levelized costs of electricity and chlorine against membrane thickness are given in Figure S3.

For a combined RED + H_2_ + Cl_2_ system, valuable insights can be obtained by considering the revenue contributions of each product as a function of different parameters. Accordingly, the revenue contribution dependence on permselectivity, number of cell pairs, and membrane thickness is plotted in Figure 5d. An increase in permselectivity results in an increase in both the voltage and current. Thus, the increase in electricity production is more pronounced than hydrogen or chlorine production. This translates to a higher revenue contribution by electricity. Increasing the number of cell pairs increases the proportion of electricity output and, hence, lower LCOE while higher LCH and LCCl (Figure 5b). Reducing the membrane thickness increases the output from the system. Even though the revenue obtained from thin membranes is higher than that from thick membranes (Supporting Information, Table S1), the relative contribution of products remains the same. Only the number of cell pairs affects the relative contributions of electricity versus hydrogen and chlorine. The current economics of the system (performed at maximum power density) reveals the relative contributions of products and the additional revenue obtained by producing chlorine under various scenarios. Since the economic value of chlorine is greater than oxygen, the current RED + H_2_ + Cl_2_ system can yield more positive economic cases than RED + H_2_ + O_2_ systems.

### Sensitivity Analysis

**Figure 6:**
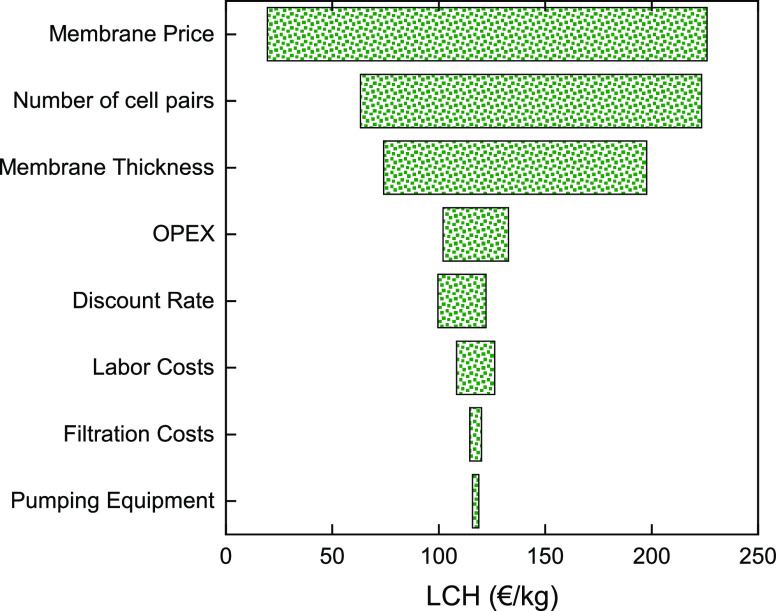
Sensitivity analysis for LCH against the top influencing parameters.

Figure 6 presents the influential parameters of a sensitivity analysis against the LCH using the default values from Table 2. The sensitivity analyses for electricity and chlorine are given in the Supporting Information, Figures S4 and S5, respectively. Each parameter is varied in between the range reported in Table 2. It can be clearly seen that the membrane price has the greatest influence on the LCH followed by the number of cell pairs and the membrane thickness. It must be noted that, even though reducing the membrane thickness or decreasing the number of cell pairs favors hydrogen production, thus reducing the costs, it is the reduction in membrane prices that brings the cost of products closer to the current market values. OPEX costs and the discount rate have a moderate impact on the levelized cost of hydrogen. As OPEX costs primarily include the costs for pre-treatment, periodic cleaning/maintenance of the membranes, and equipment, they strongly depend on the specific site/location of the plant, and hence, the optimization of these parameters must be performed carefully during designing of the process to ensure cost-effective operation. A reduction in LCH at low discount rates indicates that the rate of return on this process is low. This is followed by the labor costs, which then influence the LCH. It should be noted that labor costs only include the actual building of the setup and exclude other costs such as installation of filtration and pumping equipment. The pumping and filtration equipment costs appear to have the least impact on the LCH.

The sensitivity analysis indicates that the membrane-related parameters (thickness and costs) and number of cell pairs remain the key influencing parameters for hydrogen production. The membrane performance (resistance and apparent permselectivity) and membrane price are exactly the same parameters that are most influential for the feasibility of RED for electricity only. Hence, the commercialization of this technology depends mostly on the development of low cost, high performing membranes.

Given the imperfect membrane selectivity and the presence of chloride-containing feed streams (such as brine or seawater), chlorine evolution at the anode is inevitable in RED systems operating at industrial scales. Since the economic value of both hydrogen and chlorine is in the same order of magnitude as the electricity produced by RED systems fed with brine and seawater, selling hydrogen and chlorine as a product seems to be a logical step to improve the techno-economics of RED systems. This can be realized since the current RED system does not saturate either the hydrogen or the chlorine market (see Table S2), and the RED system produces more products than other commercial processes. While calculating the results at the maximum net power density, the technical aspects demonstrated that the membrane thickness and the permselectivity primarily determine the net power density of the system, while the ratio between electrical power and hydrogen equivalent power is determined by the number of cell pairs. In terms of economic parameters, the membrane price, thickness, and the number of cell pairs are strongest levers in determining the levelized costs of products. However, when using default values (which represent the present state of the system), the levelized costs of products are approximately 1–2 orders of magnitude higher than the current market prices, and none of the individual improvements, such as the membrane price, thickness, or permselectivity, can achieve competitive prices for the products from the proposed system. However, when combining a low membrane price (5 €/m^2^), thin membrane (40 μm), and a high permselectivity (95%), the levelized cost of hydrogen becomes competitive (<4 €/kg). This implies that the focus should include membrane technology developments, both in terms of manufacturing of highly selective and thin membranes and cost reduction to make the system competitive with existing commercial processes. We also conclude that accepting hydrogen evolution as a product in RED boosts the total energy carrier output significantly and that hydrogen and chlorine contribute for roughly half of the total revenue, at least when RED is fed with brine and seawater. This emphasizes the high potential of combined RED and hydrogen as this remains the only system that produces green electricity, hydrogen, and chlorine simultaneously.

## Supporting Information Available

Maximum obtainable current density; levelized cost of electricity (LCOE) and levelized cost of chlorine (LCCl) against the membrane price and membrane thickness; sensitivity analysis for LCOE and LCCl; annual revenue contribution of products under various conditions; annual production of electricity, hydrogen, and chlorine under various conditions (PDF)
