# Supplementary material for: Feasibility of Producing Electricity, Hydrogen, and Chlorine via Reverse Electrodialysis
Source: Environ Sci Technol. 2022 Oct 18;56(22):16062–72. doi: 10.1021/acs.est.2c03407 (PMC9671052; doi:10.1021/acs.est.2c03407)
Supplement: Supplementary file 1 — es2c03407_si_001.pdf [file es2c03407_si_001.pdf]

Supporting Information

Feasibility of Producing Electricity, Hydrogen and Chlorine via Reverse Electrodialysis

*Ameya Ranade†, Kaustub Singh†, Alessandro Tamburini‡, Giorgio Micale‡, David A. Vermaas†**

†Department of Chemical Engineering, Delft University of Technology, Van der Maasweg 9, 2629 HZ Delft, Netherlands

‡Dipartimento di Ingegneria, Università degli Studi di Palermo, viale delle Scienze Ed. 6, 90128 Palermo, Italy

(*E-mail: D.A.Vermaas@tudelft.nl)

Supporting Information Content

- 4 Pages
- 5 Figures
- 2 Tables

Contents

1. Maximum obtainable current Density (A/m^2), calculated by dividing the electromotive force by the resistance of a cell pair and is plotted with the concentrate stream on x axis and diluate stream on y axis.
2. a) Levelized Cost of Electricity (LCOE) and b) Levelized Cost of Chlorine (LCCl) plotted against membrane price
3. a) Levelized Cost of Electricity (LCOE) and b) Levelized Cost of Chlorine (LCCl) plotted against membrane thickness
4. Sensitivity Analysis for LCOE against the top influencing parameters
5. Sensitivity Analysis for LCCl against the top influencing parameters
6. Annual Revenue Contributions of products at various conditions
7. Annual production of electricity, hydrogen and chlorine at various conditions

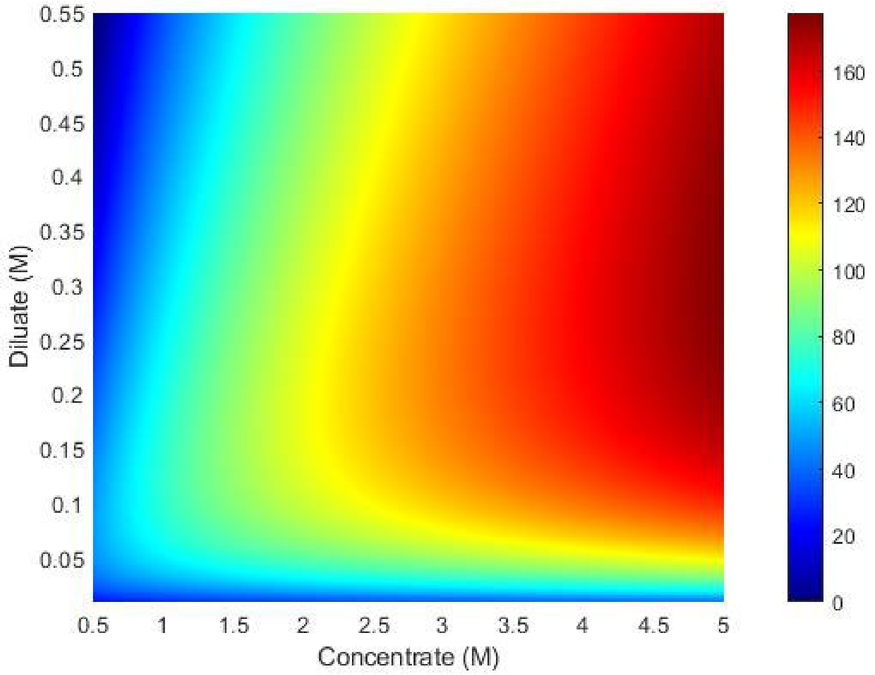

Figure S1. Maximum obtainable current Density (A/m^2), calculated by dividing the electromotive force by the resistance of a cell pair and is plotted with the concentrate stream on x axis and diluate stream on y axis. The plot considers the default values from Table 2 in the main text

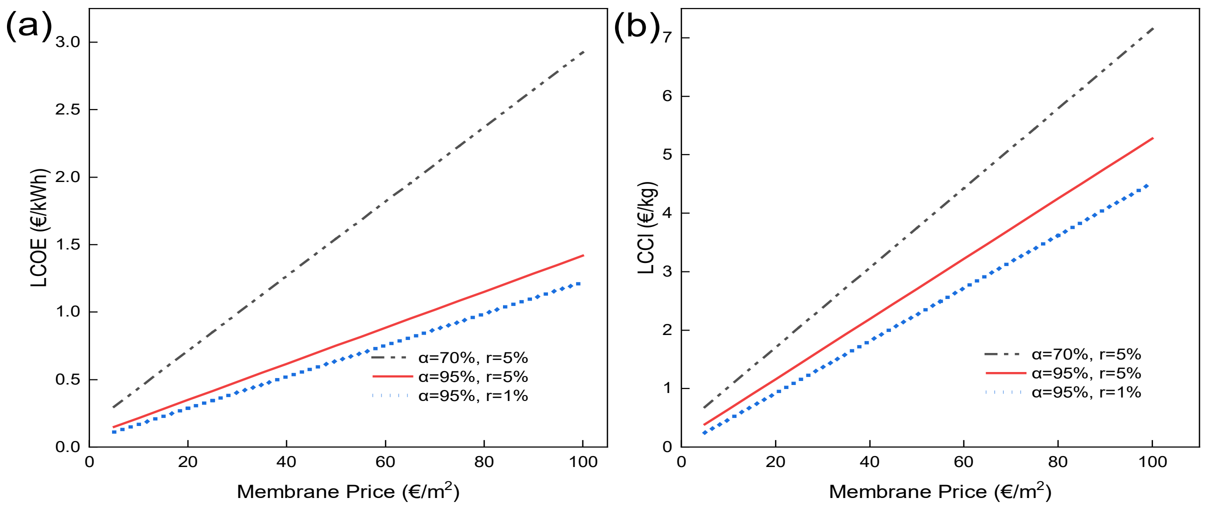

Figure S2. a) Levelized Cost of Electricity (LCOE) and b) Levelized Cost of Chlorine (LCCl) plotted against membrane price

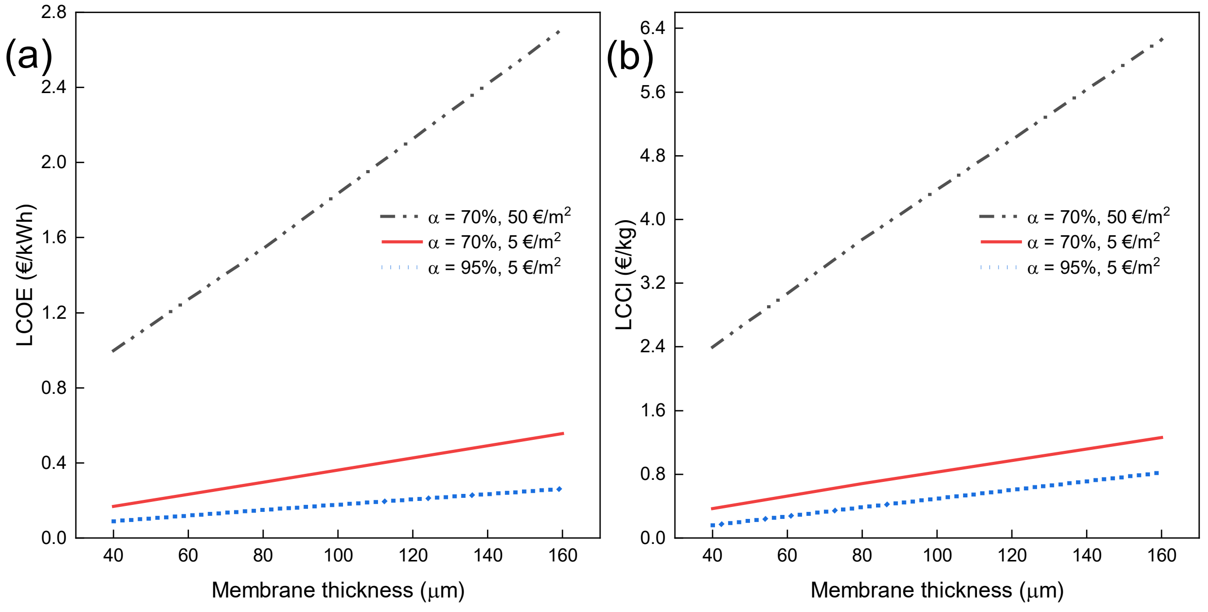

Figure S3. a) Levelized Cost of Electricity (LCOE) and b) Levelized Cost of Chlorine (LCCl) plotted against membrane thickness

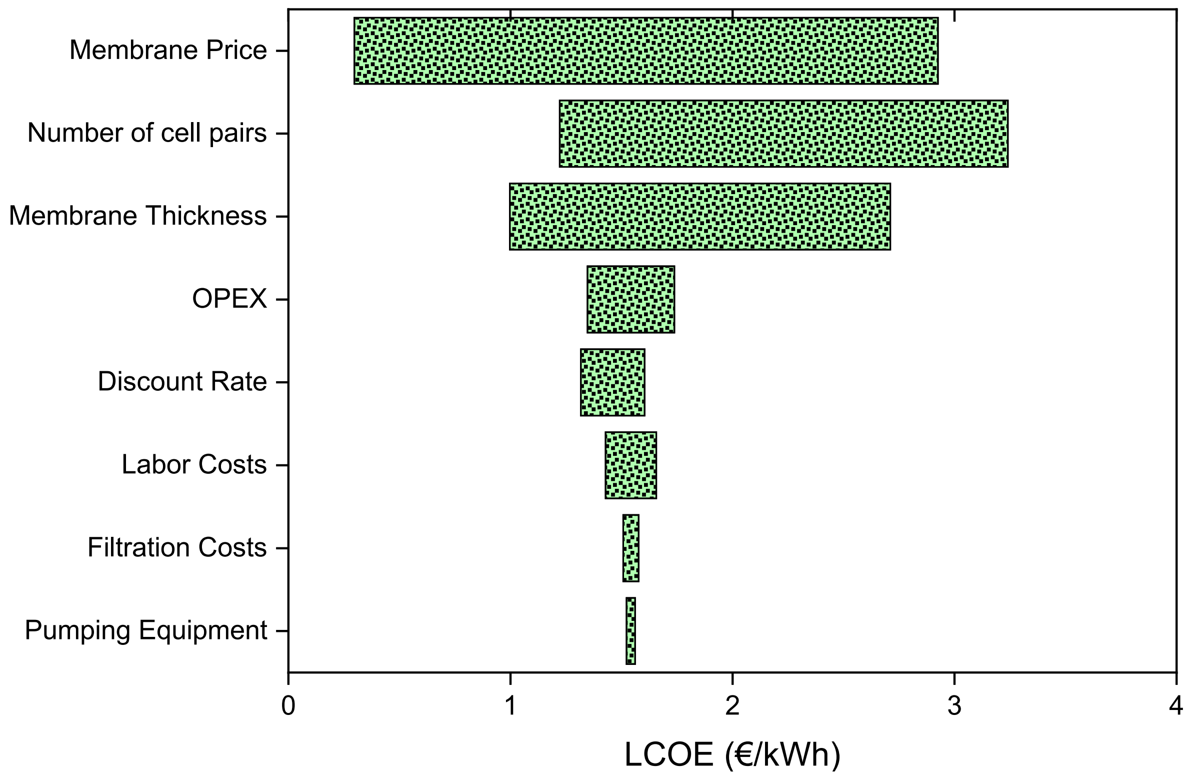

Figure S4. Sensitivity Analysis for LCOE against the top influencing parameters

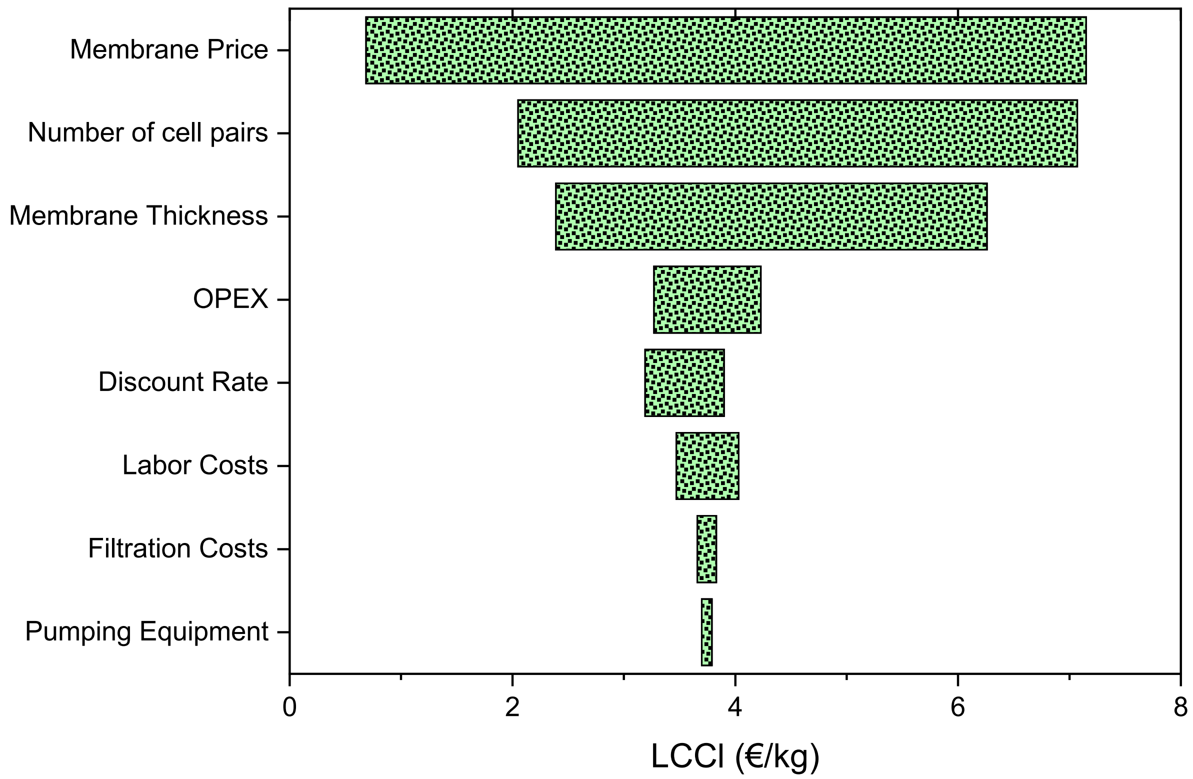

Figure S5. Sensitivity Analysis for LCCl against the top influencing parameters

Table S1. *Annual Revenue Contributions of products at various conditions*

	Permselectivity		Cell pairs		Membrane Thickness	
	α = 70%	α = 95%	50	200	40 μm	160 μm
Electricity	€ 10890	€ 22539	€ 5119	€ 13841	€ 16360	€ 6295
Hydrogen	€ 5544	€ 7327	€ 10852	€ 2831	€ 8378	€ 3395
Chlorine	€ 8856	€ 11705	€ 17336	€ 4522	€ 13384	€ 5423
**Total**	**€ 25290**	**€ 41572**	**€ 33306**	**€ 21194**	**€ 38123**	**€ 15113**

Table S2. *Annual production of electricity, hydrogen and chlorine at various conditions*

	Permselectivity		Cell pairs		Membrane Thickness		Commercial Production (Annual)
	α = 70%	α = 95%	50	200	40 μm	160 μm	
Electricity (MWh)	109	225	51	138	164	63	
Hydrogen (ton)	1.39	1.83	2.71	0.71	2.09	0.85	≈255 [1]
Chlorine (ton)	44.28	58.53	86.68	22.61	66.92	27.12	≈123000 [2]

References

(1) Buttler, A.; Spliethoff, H. Current Status of Water Electrolysis for Energy Storage, Grid Balancing and Sector Coupling via Power-to-Gas and Power-to-Liquids: A Review. *Renew. Sustain. Energy Rev.* **2018**, *82*, 2440–2454. https://doi.org/10.1016/J.RSER.2017.09.003.

(2) Chlor-alkali Industry Review 2019-2020 https://www.chlorineindustryreview.com/wp-content/uploads/2021/11/Chlor-Alkali-Industry-Review-2020-2021.pdf (accessed Sep 30, 2021).
